# Neural basis of linguistic factors involved in thought: an fMRI study with native signers

**DOI:** 10.3389/fpsyg.2025.1582136

**Published:** 2025-08-14

**Authors:** Rimi Hino, Keita Umejima, Natsumi Wada, Wataru Takei, Yoshiko Kawasaki, Kuniyoshi L. Sakai

**Affiliations:** ^1^Department of Basic Science, Graduate School of Arts and Sciences, The University of Tokyo, Tokyo, Japan; ^2^Department of Neurology, Graduate School of Medicine, The University of Tokyo, Tokyo, Japan; ^3^NPO Comekko, Osaka, Japan; ^4^Faculty of Education, Institute of Human and Social Sciences, Kanazawa University, Ishikawa, Japan; ^5^Graduate School of Human Development and Environment, Kobe University, Hyogo, Japan

**Keywords:** language, thought, sign language, fMRI, frontal cortex

## Abstract

Linguistic factors are critically involved in our conscious thinking processes, but neuroscientific evidence of their involvement is scant. To examine commonalities that underlie reasoning and language tasks, we prepared illustrative quizzes under five conditions in a Reasoning task: Context, Fill-in, Rotation, Sequence, and Analogy. These conditions differentially involved linguistic factors of the recursive, propositional, and clausal, as well as non-linguistic factors. We also used story videos in Japanese Sign Language (JSL) in a Sign task as a language comprehension task. Brain activations measured with functional magnetic resonance imaging (fMRI) were examined for native JSL signers, with the following results. First, in the comparison of the Context condition with the Fill-in condition, which controlled non-linguistic factors, multiple bilateral regions were activated, including language areas such as the left lateral premotor cortex (L. LPMC) and left inferior frontal gyrus (L. IFG). By using conjunction and region of interest analyses, we clarified two distinct systems, which were differentially recruited under the Sequence and Analogy conditions: the *recursive* system (L. LPMC/dorsal IFG and right LPMC) and the *propositional* system (L. IFG), respectively. Secondly, during the Sign task, we identified activations in the L. LPMC, L. IFG, and other temporal regions. Moreover, by focusing on the contextual comprehension processes in the Sign task, we found that the L. IFG and bilateral posterior temporal gyri (pTG) were commonly activated between the Sign task and Context condition. Thirdly, in the bilateral pTG, activations were selective only under the Context condition and not under the other four conditions, confirming its role as the *clausal* system. We thus successfully identified three critical systems for both language and thought processes.

## Introduction

In our conscious thinking processes, linguistic processes support the framework of logical reasoning, thus playing crucial roles. In their theory of the basic system design of language, Berwick et al. (2013) proposed that every linguistic expression is assigned an interpretation at a conceptual-intentional interface, which connects “mental expressions to semantic-pragmatic interpretation, reasoning, planning, and other activities of the internalized ‘mental world’.” However, neuroscientific evidence of underlying mechanisms of the relationships between language and thought remains inconclusive. Three crucial issues impeding investigation of these relationships can be pointed out. First, the commonly used neuropsychological assessments, such as the Wechsler Adult Intelligence Scale (WAIS) (Wechsler, 1997, 2008), are categorized as verbal or non-verbal according to the test stimuli, without considering whether linguistic processes are internally associated or not. For example, the picture arrangement test in WAIS has often been included with the “perceptual organization” factor but not with the “verbal comprehension” factor (Allen and Barchard, 2009), even though linguistic processes should be internally involved in story comprehension and construction. Secondly, while some researchers assume “verbal components” in tests with non-verbal stimuli (Lezak et al., 2012), distinct components of linguistic structures have not been assessed in reasoning tests. Those components are actually inseparable from thinking processes. Indeed, “The concept ‘thought’ fades into obscurity insofar as we depart from linguistically formulated thoughts” (Chomsky, 2021). Thirdly, various types or modules of reasoning have been examined in *different* test formats or settings. The Raven’s Progressive Matrices (Raven and Raven, 2003) are commonly used intelligence tests with a unified format of questions, but within this test, the modules of reasoning are severely limited. In order to assess whether the essential processes of language also substantiate thought, it is necessary to create assessment tests with multiple conditions that include a combination of linguistic components, but in a unified format.

In essence, thinking processes or thought crucially involve at least some of following linguistic components. First, the *recursive* denotes that a structure is embedded within another structure, where this embedding operation is applied in a stepwise manner; this property underlies any linguistic expression (Bar-Hillel, 1953; Freidin, 2014). This factor also provides the basis of numbers and arithmetic (Chomsky, 2021), independently from semantics. The second and third factors are the *propositional* and *clausal*, which are related to semantics (Chomsky, 2024). “Several categories of thought are relevant to language structure and use. One category is *propositional*: basic theta-structure [NB: theta-structure means structure of a predicate and its arguments with semantic roles]. A second is *clausal*: force- and information-related (interrogative, topic, focus,…) [NB: force and information establish the event structure that a sentence depicts]. The familiar property of duality of semantics [is this]” (Chomsky, 2024). These factors of recursive, propositional, and clausal are required prior to the conceptual-intentional interface, whereas “our internal speech is very likely fragments of re-internalized external speech” (Chomsky and McGilvray, 2012), i.e., comprising pragmatic language use independent of these factors. We thus designed a Reasoning task that highlighted the requirement of these *linguistic factors*: recursive, propositional, and clausal.

**Figure 1:**
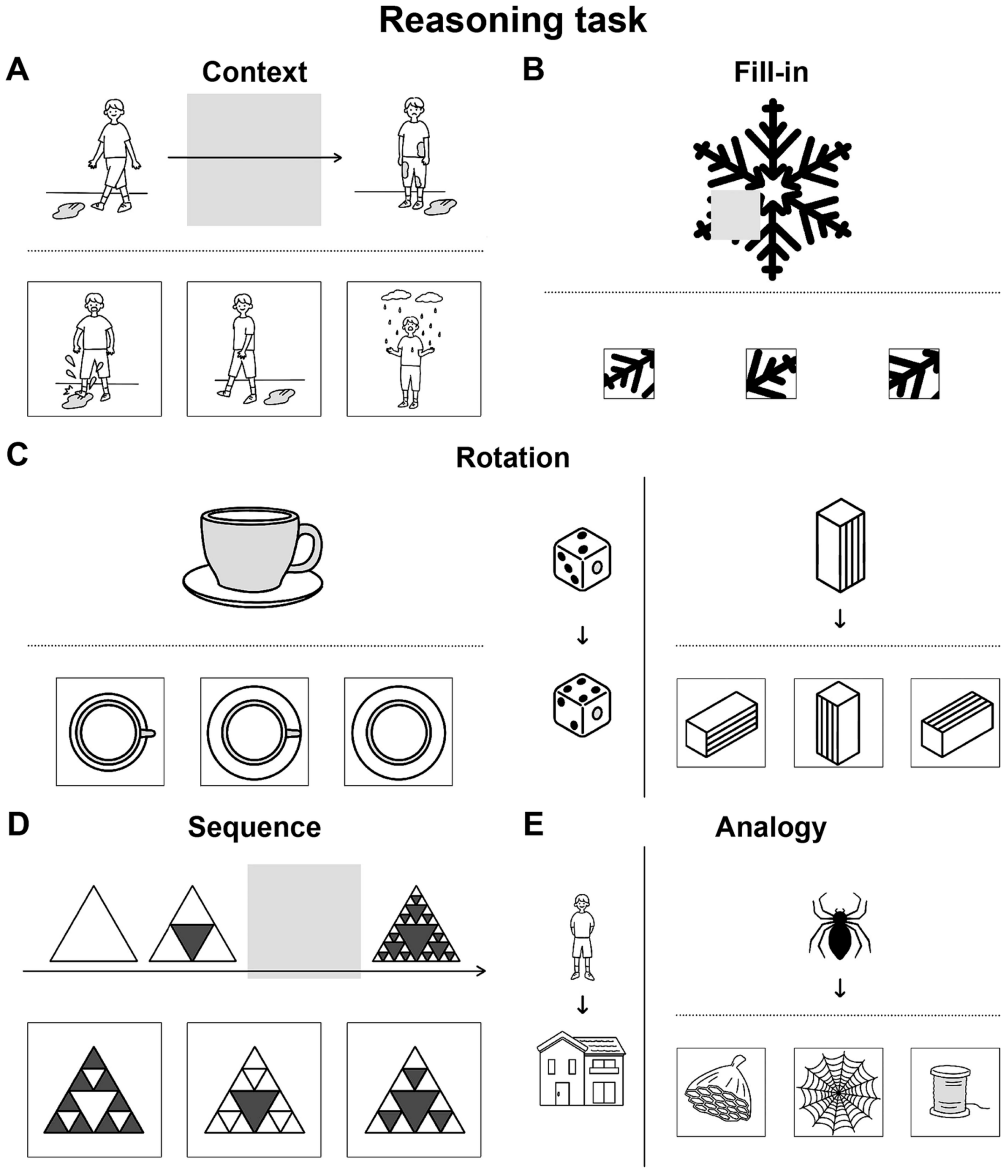
Illustrative quizzes to examine reasoning through a visual mode. We prepared quizzes under five conditions specially designed for our Reasoning task, which were visually presented without using letters. From three given choices in the lower row, participants chose the most appropriate. **(A)** An example of a quiz under the *Context* condition, in which a correct picture (the leftmost one in this case) completed a three-phase story given in the upper row. **(B)** An example of a quiz under the *Fill-in* condition, in which a correct portion (rightmost) completed a whole. **(C)** Representative quizzes under the *Rotation* condition. The quiz at left is self-contained, and the quiz at right has a reference/guide (in this case, dice) appended on its left side. Among the three choices for each, the center and leftmost ones matched the original figures: a cup/saucer and square pillar, respectively. **(D)** An example under the *Sequence* condition, in which the correct diagram (right) completed a three- or four-phase sequence. **(E)** An example under the *Analogy* condition, with a reference/guide appended at left. The correct picture (center) had a relationship with the top object that was analogous to the relationship between the objects in the reference. Note that the formats of the problems were partially shared and controlled by multiple conditions, such that the following conditions shared the same format: Context and Sequence, Fill-in and Rotation (self-contained), and Rotation (with a reference) and Analogy. For a summary of the cognitive factors involved in each condition, see Table 1.

We newly devised illustrative quizzes for a Reasoning task, where each trial consisted of a question/cue and three choices of possible answers in a unified format. The quizzes were categorized into five conditions: Context, Fill-in, Rotation, Sequence, and Analogy. To ensure that participants actually employed specific operations, we made the Context condition require all of the three linguistic factors, the Fill-in condition require none of them, and the other conditions require portions of them (Table 1). The *Context* condition tested completion of a story (Figure 1A), where individual scenes comprise a recursive sequence of events that are propositional and clausal. In contrast, the *Fill-in* condition, which tested completion of a whole picture (Figure 1B), required patterning (i.e., repeating patterns), rotating, and scaling objects as non-linguistic factors (see Table 1). This Fill-in condition thus served as the most suitable control condition for our experiments. This condition also controlled internal speech, which was equally included under all conditions we tested. The *Rotation* condition, which tested mental rotation of familiar objects or simple figures (Figure 1C), required two linguistic factors of the recursive and propositional, as well as two non-linguistic factors of rotating and scaling. The factor of the recursive is partially required, because three-dimensional objects can be explicitly rotated in a stepwise manner (e.g., 180° = 90° × 2 for a flipped case we tested), as partially supported by cognitive experiments (Shepard and Metzler, 1971). In addition, the description of rotating which object to what extent is evidently propositional, and partially required. The *Sequence* condition tested completion of a sequential pattern of diagrams (Figure 1D), requiring the following factors: recursive, patterning, and scaling, where recursive applications of rules were mandatory. Finally, the Analogy condition, which tested matching of analogous relationships (Figure 1E), fully required the propositional description of their relations, such that the relation between two objects was represented by a predicate, where objects served its arguments. The recursive might be useful for forming more extensive relations like self-embedding, but that factor was not required for associative relations we tested. As shown in Table 1, the contributions of linguistic and non-linguistic factors are clearly different among the five conditions in the Reasoning task.

**Table 1:** Linguistic and non-linguistic factors required under each condition in the Reasoning task.

Conditions	Linguistic			Non-linguistic		
	Recursive	Propositional	Clausal	Patterning	Rotating	Scaling
Context	++	++	++	−	−	−
Fill-in	−	−	−	++	++	++
Rotation	+	+	−	−	++	+
Sequence	++	−	−	+	−	++
Analogy	−	++	−	−	−	−

The estimated linguistic and non-linguistic factors are shown for each condition in the Reasoning task. ++: fully required; +: partially required; −: not required.

The present study aims at clarifying how such a sharp delineation is reflected in brain activations under these five conditions, searching for cortical regions that mirror the experimentally plausible contributions of the linguistic factors. In deducing a causal link from specific factors to brain activations, our hypothesis-based approach is alternative to behaviorally or neuropsychologically validated induction, thereby avoiding often problematic reverse inference. Even though linguistic factors are abstract, we minimized the number of factors following “Ockham’s razor,” providing a simpler model and account for thinking processes we tested.

**Figure 2:**
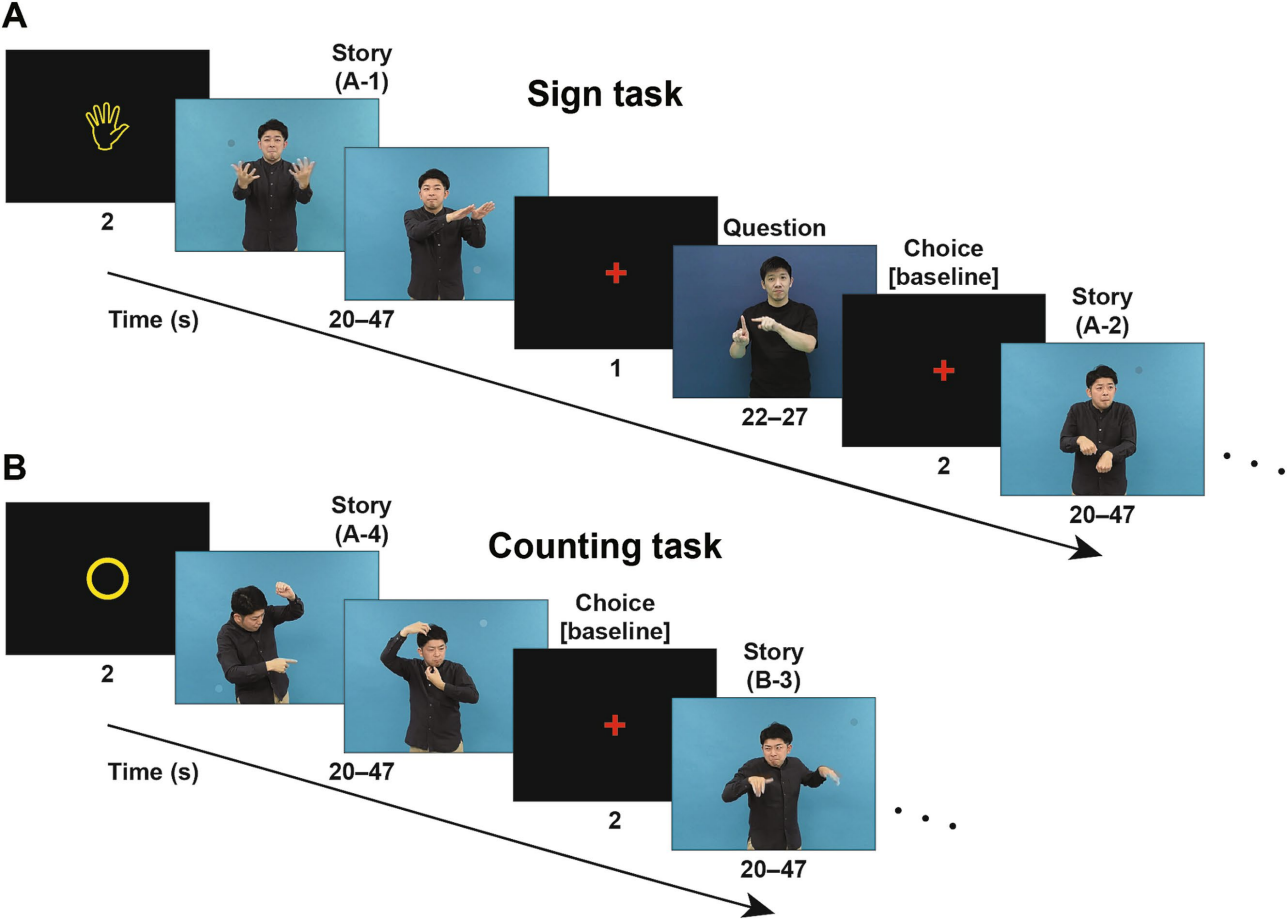
Signed sentences of stories to examine language through a visual mode. **(A)** An example trial sequence in the Sign task (starting with a “hand” symbol), where a scene of a story video (a “Story” event) in Japanese Sign Language (JSL) was presented. On the same video, one to three small circles colored similarly to their surroundings appeared at random positions and times as distractors (note the circle in each screenshot); these random circles were used in another control task (see below). A question video (“Question”) about the scene, followed by three possible answers, was subsequently presented in JSL, and the participants chose the most appropriate of three answers (“Choice”). In a scanning run, four scenes of signs comprising a single story (one of the stories A, B, and C) were presented in the original order (e.g., A-1, A-2, A-3, and A-4). **(B)** An example trial sequence in the Counting task (starting with a “circle” symbol). In a scanning run, the same Story videos were used as in the Sign task, but two different stories were mixed and individual scenes of signs were presented in a randomized order (e.g., A-4, B-3, A-2, and B-1). Upon the completion of each scene, the participants reported the number of above-mentioned circles appearing in that scene. For brain activation analyses, we obtained activations during the fMRI events of Choice as a baseline in each run.

Along with the Reasoning task via pictures alone, we needed a language comprehension task that could complement active reasoning and thinking processes, ideally without using letters or speech sounds. Sign languages satisfied this purpose with full linguistic properties (Sandler and Lillo-Martin, 2006; Baker et al., 2016); we thus prepared story videos and a Sign task in Japanese Sign Language (JSL) for hearing and deaf signers (Figure 2A). Note that sign language may potentially evoke phonological representations for hearing signers, but we have previously established that their functional organization is essentially similar to that of deaf signers (Sakai et al., 2005). After watching a scene in JSL, participants were presented with a video question in JSL, followed by three choices of possible answers. As a control, participants were presented with randomly appearing circles in a Counting task, while individual story scenes were simultaneously presented in a randomized order (Figure 2B). The use of counting is external to language, i.e., natural languages do not have a grammar based on counting, such as inserting a word at the third position of a sentence (Musso et al., 2003; Fukui, 2015). We thus used the Sign task to directly identify the critical regions during language processes, and to compare activated regions with those in the Reasoning task.

The inferior frontal gyrus (IFG, or third frontal gyrus [F3]) in the left hemisphere is known as one of the language areas responsible for specific linguistic processes. The left lateral premotor cortex (L. LPMC, just dorsal to F3) and opercular/triangular parts of the left F3 (L. F3op/F3t) have been identified as “grammar centers” related to syntactic structures, whereas the triangular/orbital parts of the left F3 (L. F3t/F3O) are known as the center for sentence comprehension (Sakai, 2005). Moreover, especially during grammatical processing in a second language, the right lateral premotor cortex (R. LPMC) and opercular/triangular/orbital parts of the right F3 (R. F3op/F3t/F3O) have been reported to play supportive roles (Sakai et al., 2004; Tatsuno and Sakai, 2005). On the other hand, in a study using the Pyramids and Palm Trees Test (Howard and Patterson, 1992) as a semantic test, activations in the R. LPMC, R. F3O, and left posterior temporal gyrus (L. pTG) have been observed for the young healthy participants (McGeown et al., 2009). We hypothesized that the linguistic factors of the recursive, propositional, and clausal are selectively represented in these distinct regions. Our results could provide a foundation for a neuroscientific model of the relationship between language and thought.

## Materials and methods

### Participants

We recruited 19 native signers of JSL, who had been exposed to JSL since birth. Laterality quotients (LQ) were evaluated by the Edinburgh inventory (Oldfield, 1971), and 1 participant was excluded from analyses due to left-handedness (i.e., negative LQ). Among the remaining 18 participants (11 females; age: 30.0 ± 7.3 years; LQ: 89 ± 22 [mean ± SD]), 12 were Deaf signers who were also able to communicate in written Japanese (JPN), and the other 6 were hearing signers of JSL/JPN bilinguals with Deaf parents. Throughout experiments for the Deaf participants, conversations between experimenters and participants were translated into JSL/JPN by professional sign language interpreters, either face-to-face or via a video system. For instructions outside the scanner, sheets in JPN were provided for task instructions.

All of the participants provided their written informed consent to participate in this study, after the nature and possible consequences of the study were explained. Approval of these experiments was obtained from the institutional review board of the University of Tokyo, Komaba Campus (approval no. 755–2). All research studies were performed in accordance with the Declaration of Helsinki, the Singapore Statement on Research Integrity, and the relevant guidelines/regulations in Japan (the Science Council of Japan and the Japan Society for the Promotion of Science).

### Stimuli

For the Reasoning task (see the Introduction), we newly prepared 50 illustrative quizzes categorized into five conditions (10 quizzes for each): Context, Fill-in, Rotation, Sequence, and Analogy (Figure 1). Without using letters, a question or cue was presented in the upper row, together with three choices of possible answers in the lower row. These three choices were graded according to major and/or minor criteria required by each condition: a best choice (3 points) that satisfied both criteria, a second-best choice (1 point) that met a major criterion alone, and a wrong choice (0 points) that met a minor criterion alone. The three choices were in random positions, but always presented with the question/cue; the involvement of short-term memory was thus excluded.

For example, under the Context condition, the participants had to complete a three-phase story by choosing a correct scene for the second phase (see Figure 1A). Choices consistent with the first phase satisfied a major criterion, while those consistent with the third phase satisfied a minor criterion, since the former follows the normal direction of the story. The criteria under the other conditions would be self-explanatory from the examples shown in Figures 1B–E. For the estimation of accuracy rates, we summed up the above-mentioned points.

For the Sign and Counting tasks (Figure 2), we prepared three consecutive stories in JSL, each of which was split into four scenes (20–47 s each) in accordance with natural context changes. The video images were in the mpeg format (29.97 fps) with 800 × 600 pixels. In the videos, small circles colored similarly to their surroundings were simultaneously presented. In each of the four scenes, one to three circles were presented for 1 s each in random positions at random times.

For each scene in the Sign task, we also prepared a video question in JSL, together with three possible answers in JSL as well (22–27 s). For example, the scene A-1 in Figure 2A can be briefly summarized in English as follows: “In a lush forest, a herd of elephants walks along. A parent elephant warns its child not to wander off. Later, the curious child chases a butterfly.” The accompanying question was: “What did the elephant chase after?” Possible answers were: “① a squirrel; ② a flower; and ③ a butterfly.”

When a stimulus was not presented, a small red cross was presented for eye fixation. All stimuli were presented through an eyeglass-like MRI compatible display (VisuaStim Digital; Resonance Technology Inc., Northridge, CA; resolution = 800 × 600, framerate = 60 fps), and the lens powers were adjusted for weak-sighted participants. The stimulus presentation was controlled by the Presentation software package (Neurobehavioral Systems, Berkeley, CA), which also collected behavioral data (accuracy and response times [RTs]). The participants provided their answers by pressing buttons on a response pad (HH-1x4-L; Current Designs Inc., Philadelphia, PA).

### Tasks

For the same Reasoning task, there were five conditions with different stimuli as noted above, whereas there were two different tasks of Sign and Counting with the same set of stimuli. We first analyzed behavioral and brain activation data; these analyses were conducted separately for the Reasoning task and Sign/Counting tasks. Then, we combined all of these tasks for the region of interest (ROI) analyses.

In the Reasoning task, we visually presented each quiz for 10 s, during which the participants chose the most appropriate answer by pushing one of three buttons (see Figure 1). With an interval of 1 s between quizzes, each scanning run consisted of nine quizzes under five conditions (one or two quizzes under each condition). Quizzes from the same condition were never presented consecutively. Throughout the experiment, we did not provide any verbal explanations on the condition names or details of the quizzes.

In the Sign task (Figure 2A), a run of four trials began with a visual cue of a “hand” symbol (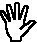). In each run, four scenes of signs, taken from the same single story, were presented in the original order. For each scene, following a pause of 1 s, we presented the corresponding video question on the contents, and then the participants chose the most appropriate answer by pushing one of three buttons within 2 s. The small colored circles were distractors, and the participants were instructed as follows: “Please concentrate on reading signs. You do not have to count circles.”

In the Counting task (Figure 2B), a run of four trials began with a cue of a “circle” symbol (○). In each run, four scenes of signs, taken from two different stories, were presented in a randomized order. For each scene, the participants chose the appropriate number of circles that appeared by pushing one of three buttons within 2 s. The scenes were distractors, and the participants were instructed as follows: “Please concentrate on counting circles. You do not have to read signs.”

The same story scene appeared once in each of the Sign and Counting tasks, always earlier in the Counting task. This was to minimize contextual flow in the Counting task. After six scanning runs in the Sign and Counting tasks (in the order of Counting–Counting–Sign–Counting–Sign–Sign), the participants took a rest for about 10 min. Then they proceeded to five runs in the Reasoning task in the same day; we obtained structural MRI data right after the final run. Before scanning, we conducted a practice trial in each of the Sign and Counting tasks, and also that under each condition in the Reasoning task.

### MRI data acquisition and analyses

The following methods conformed to the procedures published previously by our team (Ohta et al., 2017; Tanaka et al., 2017; Tanaka et al., 2019). For the MRI data acquisition, the participant was in a supine position, and her/his head was immobilized inside the radio-frequency coil. The MR scans were conducted on a 3.0 T system, GE Signa HDxt 3.0 T (GE Healthcare, Milwaukee, WI). We scanned 30 axial slices, each 3-mm thick and having a 0.5-mm gap, covering the volume range of −38.5 to +66 mm from the anterior to posterior commissure (AC-PC) line in the vertical direction, using a gradient-echo echo-planar imaging (EPI) sequence (repetition time [TR] = 2 s, echo time [TE] = 30 ms, flip angle [FA] = 78°, field of view [FOV] = 192 × 192 mm^2^, resolution = 3 × 3 mm^2^). In a single run, we obtained 56, 104–125, and 64–69 volumes in the Reasoning, Sign, and Counting tasks, respectively, all of which followed four dummy images allowing for the rise of the MR signals. After completion of the fMRI session, high-resolution T1-weighted images of the whole brain (136 axial slices, 1.0 × 1.0 × 1.0 mm^3^) were acquired with a three-dimensional fast spoiled gradient recalled acquisition in the steady state (3D FSPGR) sequence (TR = 8.5 ms, TE = 2.6 ms, FA = 25°, FOV = 256 × 256 mm^2^). These structural images were used for normalizing fMRI data.

The fMRI data were analyzed in a standard manner using the statistical parametric mapping (SPM) software package SPM12 (Wellcome Trust Center for Neuroimaging^1^) (Friston et al., 1995) implemented on MATLAB (Math Works, Natick, MA). The acquisition timing of each slice was corrected using the middle slice (the 15th slice chronologically) as a reference for the EPI data. We realigned the time-series data in multiple runs to the first volume in all runs. The realigned data were resliced every 3 mm using seventh-degree B-spline interpolation, so that each voxel of each functional image matched that of the first volume. From 1 participant, we removed a run in each of the Sign and Counting tasks, which included data with a translation of > 2 mm in any of the three directions or with a rotation of > 1.4° around any of the three axes.

After alignment to the AC-PC line, each participant’s T1-weighted structural image was coregistered to the mean functional image generated during realignment. The coregistered structural image was spatially normalized to the standard brain space as defined by the Montreal Neurological Institute (MNI), using the “unified segmentation” algorithm with light regularization, which is a generative model that combines tissue segmentation, bias correction, and spatial normalization in the inversion of a single unified model (Ashburner and Friston, 2005). After spatial normalization, the resultant deformation field was applied to the realigned functional imaging data. All normalized functional images were then smoothed by using an isotropic Gaussian kernel of 9-mm full-width at half maximum. Low-frequency noise was removed by high-pass filtering at 1/128 Hz.

In a first level analysis (i.e., the fixed-effects analysis), the hemodynamic responses of each participant under each condition (10 s per event) in the Reasoning task, as well as the variable events of Story, Question, and Choice (20–47 s, 22–27 s, and 2 s, respectively) in the Sign and Counting tasks (see Figure 2), were modeled with a boxcar function, including both correct and incorrect trials. The boxcar function was then convolved with a hemodynamic response function. To minimize the effects of head movement, the six realignment parameters obtained from preprocessing were included as a nuisance factor in a general linear model. The images under each of the five conditions in the Reasoning task, as well as those for the Sign (Story), Sign (Question), Sign (Choice), Counting (Story) [simply called “Counting” hereafter], and Counting (Choice) events, were then generated in the general linear model for each participant and used for the intersubject comparison in a second-level analysis (i.e., the random-effects analysis). Activations during Sign (Choice) and Counting (Choice) were subtracted as baselines for the Sign and Counting tasks, respectively.

A one-way repeated-measures analysis of variance (rANOVA) with *t*-tests was performed for the five conditions in the Reasoning task, as well as for the event types in the Sign and Counting tasks, including four nuisance factors (age, LQ, gender, and hearing status [i.e., deaf or hearing]). The results were thresholded at uncorrected *p* < 0.001 for the voxel level, and at *p* < 0.05 for the cluster level with family-wise error (FWE) correction across the whole brain. For each contrast, an exclusive mask of negative activations was applied (uncorrected *p* < 0.05 for the voxel level). We also conducted conjunction analyses for multiple contrasts by using the Conjunction Null Hypothesis option in SPM12, where we applied exclusive masks of activations for the [− Fill-in] and [Counting − Sign (Question)] contrasts (uncorrected *p* < 0.05 for the voxel level). For the anatomical identification of activated regions, we used the Anatomical Automatic Labeling (AAL) method^2^ (Tzourio-Mazoyer et al., 2002) and the labeled data provided by Neuromorphometrics Inc.^3^ under academic subscription.

In addition to the whole-brain analyses described above, we conducted analyses of each ROI by using the MarsBaR-toolbox^4^. We obtained ROI clusters from activations in the contrasts of Sequence − Fill-in, Analogy − Fill-in, and Sign (Story) − Counting. Following the standard usage of MarsBaR, we used those ROIs within the same participants for statistical comparisons independent from each contrast. For analyses of signal changes, as well as for analyses of behavioral data, we used the R software (ver. 4.3.2^5^).

To justify sample sizes in relation to what has been previously published, we performed a power analysis (Mumford, 2012) by using our previous work with a multiple-choice task and a similar number of trials (16 trials) (Umejima et al., 2021b). With an online estimator of Neuropower^6^, we used the whole-brain data from a group of 16 participants (Paper notebook group) and obtained average power of 93% for 18 subjects (FDR corrected *p* < 0.05), which is above the typical aim of 80% power for fMRI.

## Results

### Behavioral data reflecting different cognitive factors

**Figure 3:**
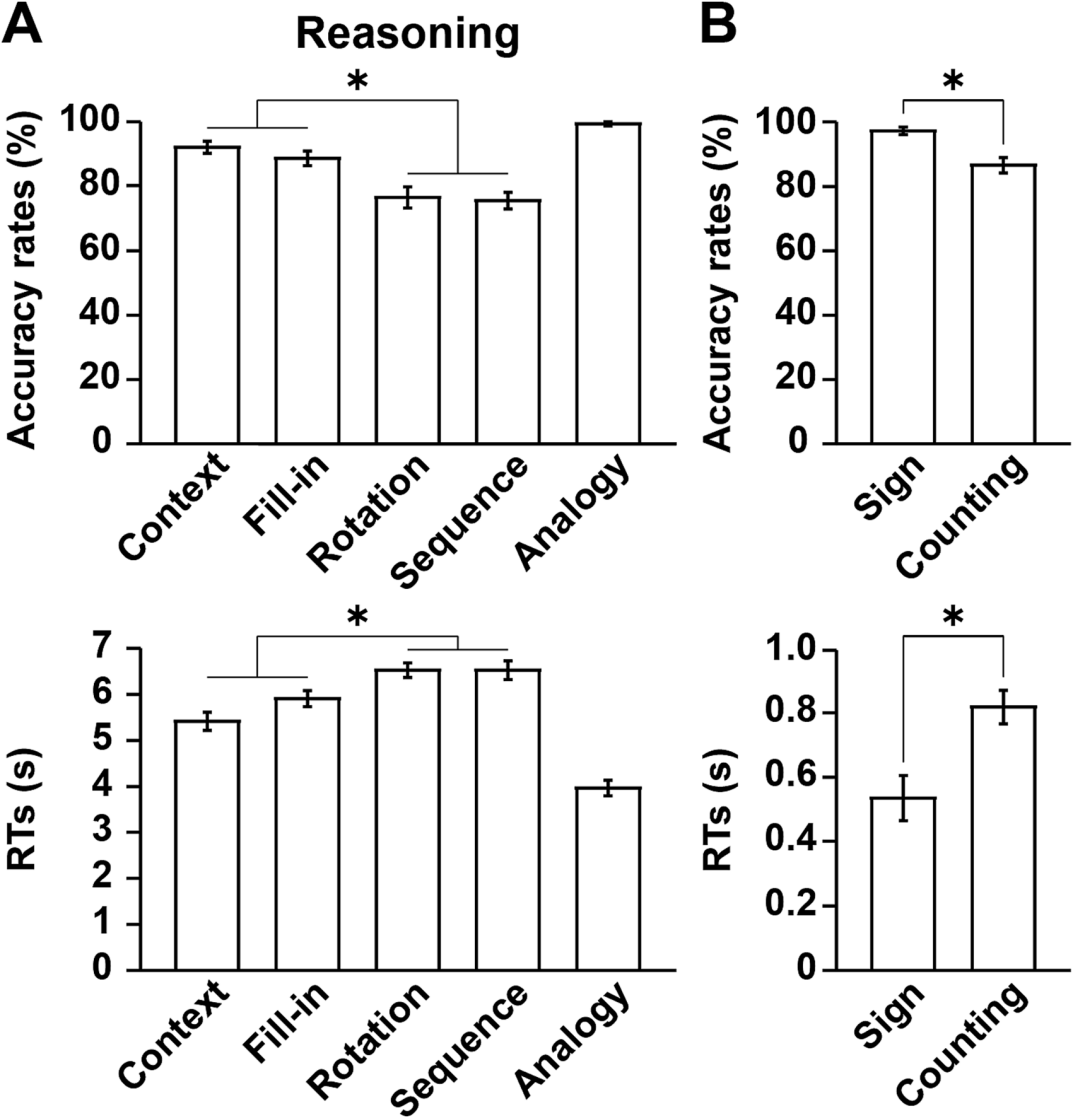
Behavioral data reflecting different cognitive factors. **(A)** Performances in the Reasoning task. Accuracy rates and response times (RTs) are shown under each condition. Note that the Analogy condition was the easiest of the five conditions. **(B)** Performances in the Sign and Counting tasks. Significantly lower accuracy rates and longer RTs were observed in the Counting task than in the Sign task. The error bars denote the SEM (standard error of the mean). *p < 0.05; n.s., not significant.

In regard to the accuracy rates and RTs, a one-way rANOVA indicated a significant main effect of condition (accuracy rates: *F*[4,68] = 26, *p* < 0.0001; RTs: *F*[4,68] = 72, *p* < 0.0001) (Figure 3A). Overall, the Analogy condition was the easiest with lowest task loads, while the Rotation and Sequence conditions were hardest. Moreover, the performances under combined Context and Fill-in were significantly easier than those under combined Rotation and Sequence (paired *t*-tests; accuracy rates: *t*[17] = 6.2, *p* < 0.0001; RTs: *t*[17] = 9.7, *p* < 0.0001). If condition-specific task difficulty existed, it would reflect the contributions of linguistic or non-linguistic factors (see Table 1), but this differential pattern of accuracy rates and RTs among the five conditions did not match any of such contributions.

With respect to the Sign and Counting tasks, significantly lower accuracy rates (Wilcoxon signed-rank test, *V* = 86, *p* = 0.003) and longer RTs (paired *t*-test, *t*[17] = 6.7, *p* < 0.0001) were observed in the Counting task than in the Sign task (Figure 3B). Because of ceiling effects for the accuracy rates in the Sign task, we used a non-parametric test.

### Differential activation patterns among conditions of the Reasoning task

**Figure 4:**
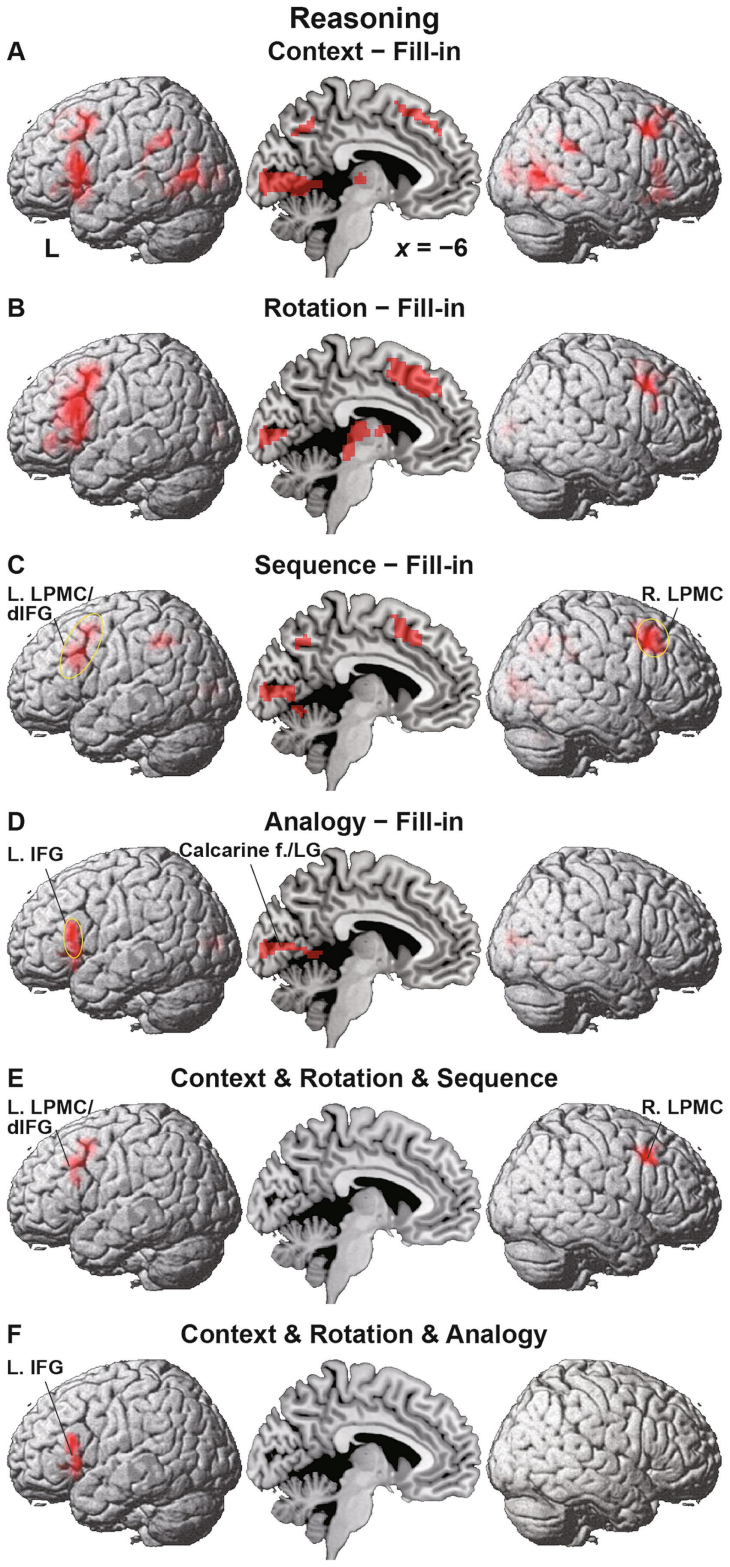
Differential activation patterns among conditions of the Reasoning task. **(A)** Activations under the Context condition, compared with those under the Fill-in condition. Significant activations were mainly observed in the bilateral frontal and temporal regions, as well as in the medial regions. **(B)** Activations under the Rotation condition, which showed enhanced left frontal activations. **(C)** Activations under the Sequence condition, which showed frontal activations localized in the left lateral premotor cortex/dorsal inferior frontal gyrus (L. LPMC/dIFG) and right lateral premotor cortex (R. LPMC). The circled clusters were used for further region of interest (ROI) analyses (see Figure 5). **(D)** Activations under the Analogy condition, which showed lateral frontal activations localized in the more ventral portion of the L. IFG. The calcarine fissure (f.) and lingual gyrus (LG) were also activated. **(E)** The result of a conjunction analysis for the Context, Rotation, and Sequence conditions. Activations were localized in the L. LPMC/dIFG and R. LPMC. **(F)** The result of a conjunction analysis for the Context, Rotation, and Analogy conditions. Activations were localized in the L. IFG alone. Significance was determined at uncorrected *p* < 0.001 for the voxel level, and at family-wise error (FWE)-corrected *p* < 0.05 for the cluster level. For each contrast, we applied an exclusive mask of activations for the [− Fill-in] (uncorrected *p* < 0.05 for the voxel level).

As mentioned in the Introduction, we adopted the Fill-in condition as the most suitable control condition. We compared activations under each condition in the Reasoning task with Fill-in, i.e., the [Context − Fill-in] contrast, etc. Under Context, we observed basically bilateral activations in the LPMC, IFG, angular/supramarginal gyri (AG/SMG), pTG, middle occipital gyri (MOG), and hippocampi, as well as medial activations in the pre-supplementary motor area (pre-SMA), precuneus, calcarine fissure, LG, and thalamus (Figure 4A and Table 2). In contrast, activations in the dorsal IFG (dIFG) were left-lateralized. It is notable that activations in the left lateral cortex included the language areas, such as the L. LPMC/dIFG, L. IFG (ventrally extended from F3op/F3t to F3O), L. AG/SMG, and L. pTG.

**Table 2:** Regions with activations observed in the Reasoning and Sign tasks.

Brain regions	BA	Side	*x*	*y*	*z*	*Z*	Voxels	Brain regions	BA	Side	*x*	*y*	*z*	*Z*	Voxels
Reasoning, Context − Fill-in								Reasoning, Context − Fill-in (Cont.)							
LPMC	6/8/9	L	−39	11	47	7.1	597	pTG	21	L	−51	−34	−10	4.6	281
dIFG	44/45	L	−36	23	35	4.8	*		21/37	L	−54	−61	5	Inf	*
F3op/F3t	44/45	L	−54	20	14	6.9	*		37/19	L	−39	−64	14	7.1	*
F3t/F3O	45/47	L	−51	20	−4	7.0	*	MOG	19	L	−36	−73	29	3.5	*
			−48	35	−10	5.5	*	Precuneus	7	M	−6	−58	41	6.6	97
LPMC	6/8/9	R	48	17	44	6.3	423	Calcarine f.	17/18	M	9	−88	11	Inf	984
			15	29	56	5.5	*				−6	−61	5	4.9	*
			30	26	50	4.8	*	LG	18/19	M	12	−64	−7	6.5	*
pre-SMA	6/8/9	M	−6	26	56	6.2	*			L	−18	−64	−10	5.2	*
			−3	44	35	4.0	*	Hippocampus/thalamus		L	−18	−31	−1	5.0	127
F3op/F3t	44/45	R	57	23	11	5.6	150	Thalamus		M	−9	−19	5	4.0	*
F3t/F3O	45/47	R	48	29	−10	6.3	*	Hippocampus		R	27	−10	−13	4.5	124
Temporal pole	38	R	54	14	−16	5.1	*	Hippocampus/thalamus		R	15	−31	−1	5.0	*
AG/SMG	39/40	L	−57	−49	29	7.8	125	Sign (Story) − Counting							
STG	22	L	−60	−34	8	4.1	*	F3op/F3t	44/45	L	−60	11	17	4.5	211
AG/SMG	39/40	R	51	−46	26	6.4	454	F3t/F3O	45/47	L	−54	20	5	4.7	*
			57	−52	41	4.2	*	pre-SMA	6/8	M	−6	20	56	4.1	104
AG	39	R	42	−67	41	4.5	*				−6	−7	59	3.5	*
pTG	21/37	R	42	−52	14	7.0	*	pTG	21	L	−60	−37	−4	4.1	430
			63	−43	−4	7.0	*		21/37	L	−57	−58	2	5.0	*
	37/19	R	54	−67	8	Inf	*		37/19	L	−48	−76	−7	3.7	*
			45	−76	−7	4.4	*	pTG	21/37	R	51	−37	2	4.8	330
MOG	19	R	45	−70	23	5.7	*				57	−58	5	4.9	*

Stereotactic coordinates (*x, y, z*) in the MNI space are shown for activation peaks of *Z* values, which were more than 16 mm apart (see Figures 4A, 6A). A region marked with an asterisk is included within the same cluster as the region in the row right above it. BA: Brodmann’s area; L: left; R: right; M: medial; AG: angular gyrus; dIFG: dorsal inferior frontal gyrus; f.: fissure; F3op/F3t/F3O: opercular/triangular/orbital parts of the F3 (IFG); LG: lingual gyrus; LPMC: lateral premotor cortex; MOG: middle occipital gyrus; pre-SMA: pre-supplementary motor area; pTG: posterior temporal gyri; SMG: supramarginal gyrus; STG: superior temporal gyrus.

Activations under Rotation were basically a subset of those under Context, but were mostly left-lateralized in the L. LPMC/dIFG, and L. IFG, together with the R. LPMC, pre-SMA, calcarine fissure, and thalamus (Figure 4B). Activations under Sequence were observed in the L. LPMC/dIFG, R. LPMC, bilateral AG/SMG, pre-SMA, calcarine fissure, and LG (Figure 4C). Under Analogy, activations were localized only in the L. IFG, calcarine fissure, and LG (Figure 4D).

It is possible that domain-general factors and cognitive demands (task difficulty, problem complexity, visual attention, spatial manipulation, action planning, etc.) partially elicited activations shown above. Based on behavioral results (see Figure 3), we noted that the Counting task vs. Sign task (the former being more difficult) provided an independent estimation of these domain-general effects and *visuospatial analyses* (aside from task-specific counting that had minimum demands), which were commonly included in the Reasoning task. In subsequent analyses, we applied an exclusive mask of activations for the [Counting − Sign (Question)] contrast (uncorrected *p* < 0.05 for the voxel level) in addition to the exclusive mask of negative activations [− Fill-in] used for the above contrasts (e.g., [Context − Fill-in]). These exclusive masks eliminated activations in the calcarine fissure and LG.

To specify common activations among some of the four conditions, we took the conjunction of Context, Rotation, and Sequence, and observed significant activations in the L. LPMC/dIFG and R. LPMC (Figure 4E). Moreover, according to the conjunction of Context, Rotation, and Analogy, significant activations were observed in the L. IFG alone, with a minimum overlap with the L. LPMC/dIFG (Figure 4F). These results indicate that the L. LPMC/dIFG and R. LPMC subserve the *recursive* system, and that the L. IFG subserves the *propositional* system (see Table 1).

### Distinct activation patterns of cortical regions in the Reasoning task

**Figure 5:**
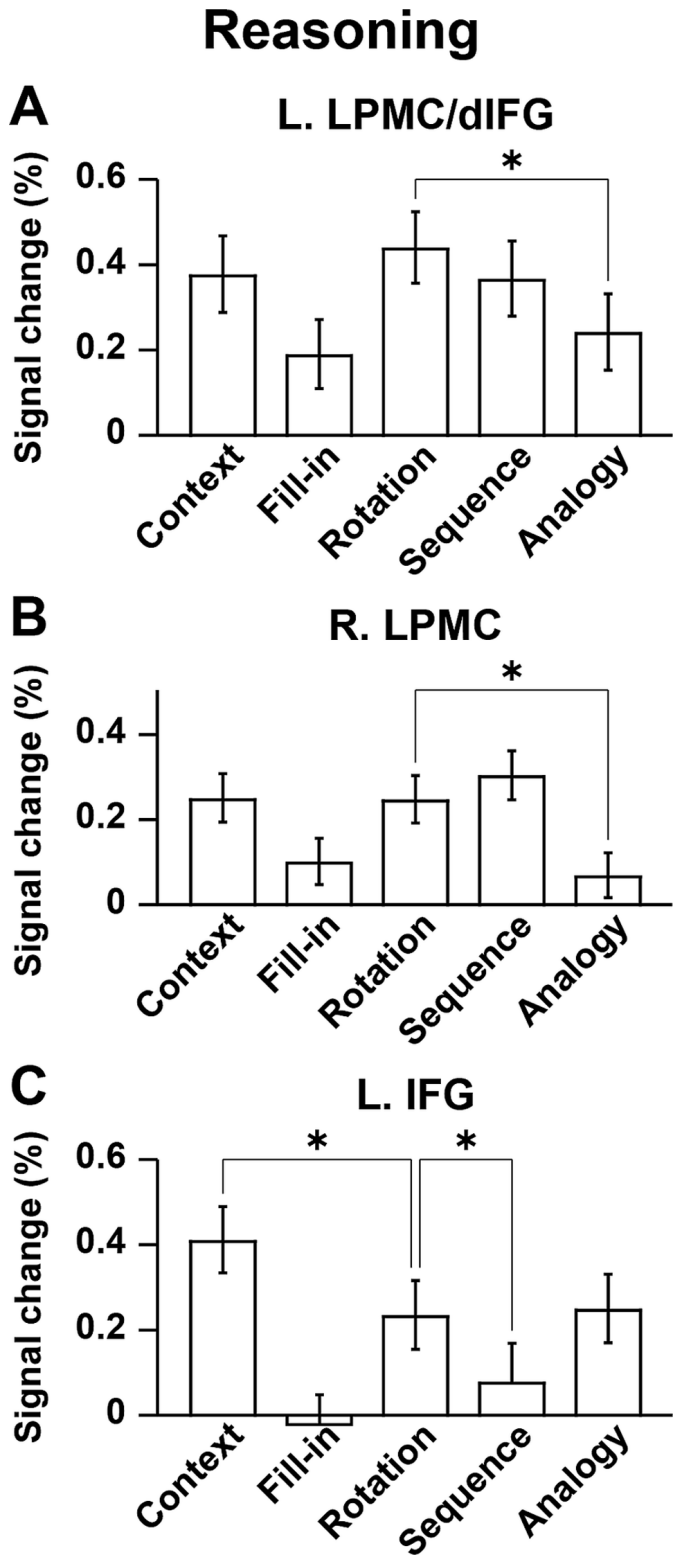
Distinct activation patterns of cortical regions in the Reasoning task. **(A)** Signal changes in the L. LPMC/dIFG. Activations were significantly lower under the Analogy condition than under the Rotation condition. **(B)** Signal changes in the R. LPMC, which replicated those of the L. LPMC/dIFG. **(C)** Signal changes in the L. IFG. The activations were significantly lower under the Sequence condition than under the Rotation condition, and significantly higher under the Context condition than under the Rotation condition. The error bars denote the SEM. *p < 0.05.

For ROI analyses in the Reasoning task, we chose the L. LPMC/dIFG, R. LPMC, and L. IFG, which were identified by the conjunction analyses shown above. The ROIs of the L. LPMC/dIFG and R. LPMC were taken from the [Sequence − Fill-in] contrast (see Figure 4C), whereas that of the L. IFG was from the [Analogy − Fill-in] contrast (see Figure 4D). We took the Rotation condition, which is independent from these contrasts, as a reference to confirm “double dissociation” between the recursive and propositional systems. In the L. LPMC/dIFG (Figure 5A) and R. LPMC (Figure 5B), activations were significantly lower under *Analogy* than under Rotation (L. LPMC/dIFG: *t*[17] = 4.9, *p* = 0.0001; R. LPMC: *t*[17] = 5.9, *p* < 0.0001). As regards the L. IFG (Figure 5C), activations were significantly lower under *Sequence* than under Rotation (*t*[17] = 3.9, *p* = 0.001). We also found that the L. IFG activations were significantly higher under *Context* than under Rotation (*t*[17] = 4.0, *p* = 0.0009), consistent with its role as the propositional system (see Table 1). Both of the two comparisons for the L. IFG activations were significant under Bonferroni correction (*p* < 0.05; *α* = 0.025).

### Localized activations identified by the Sign task

**Figure 6:**
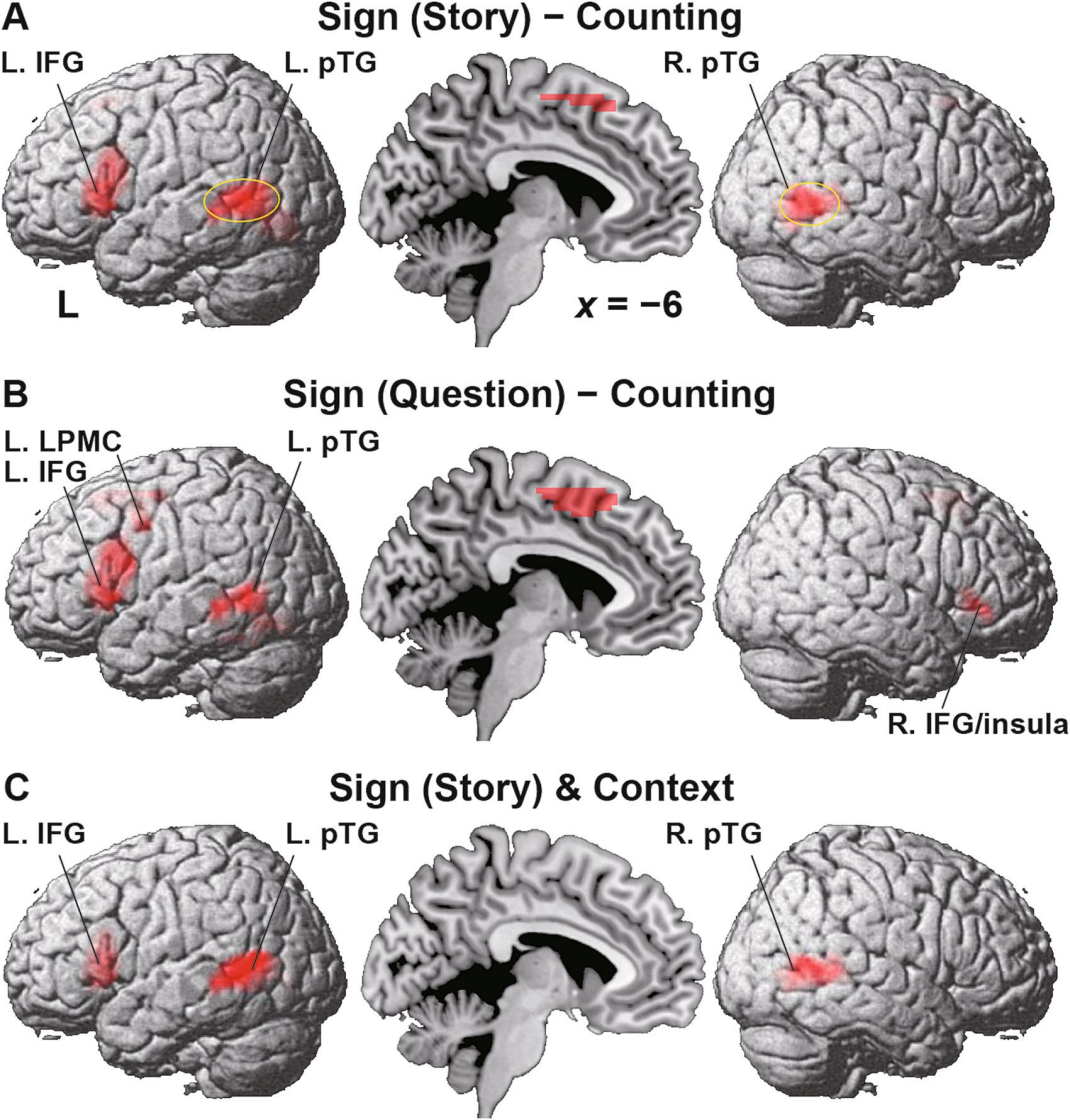
Localized activations identified by the Sign task. **(A)** Activations during the Story event in the Sign task [i.e., Sign (Story)], compared with those during the Story event in the Counting task (see Figure 2). Significant activations were observed in the L. IFG, bilateral posterior temporal gyri (pTG), and the pre-supplementary motor area. The circled clusters were used for further ROI analyses (see Figure 7). **(B)** Activations during the Sign (Question) event. In the right hemisphere, activations in the R. IFG and insula were additionally observed instead of the R. pTG. **(C)** Commonly activated regions between the Sign (Story) event and Context condition. Significant activations were localized in the L. IFG and bilateral pTG. Significance was determined at uncorrected *p* < 0.001 for the voxel level, and at FWE-corrected *p* < 0.05 for the cluster level. For each contrast of **(A,B)**, an exclusive mask (one-sample *t*-test, uncorrected *p* < 0.05) of negative activations during Counting was applied.

Regarding the Sign and Counting tasks, we compared activations during individual Story events, i.e., the [Sign (Story) − Counting] contrast, focusing on contextual comprehension processes. Significant activations were observed in the L. IFG (including the F3op/F3t/F3O), bilateral pTG, and pre-SMA (Figure 6A and Table 2). In the contrast of [Sign (Question) − Counting], which focuses on the active reasoning and thinking processes, activations in the left hemisphere were found in the L. IFG, and L. pTG, which was consistent with Sign (Story), as well as in an additional region of the L. LPMC (Figure 6B). In the right hemisphere, instead of the R. pTG, the R. IFG and insula [local maxima: (*x*, *y*, *z*) = (57, 26, 8), (48, 35, −7), (27, 26, −1)] were activated. It is notable that all of these regions, except the right insula, were also activated under the Context condition in the *Reasoning* task (see Table 2). Moreover, by applying an inclusive mask of the activated regions in the [Sign (Story) − Counting] contrast, activations under Context were localized in the L. IFG and bilateral pTG (Figure 6C), indicating their crucial roles in these common processes.

**Figure 7:**
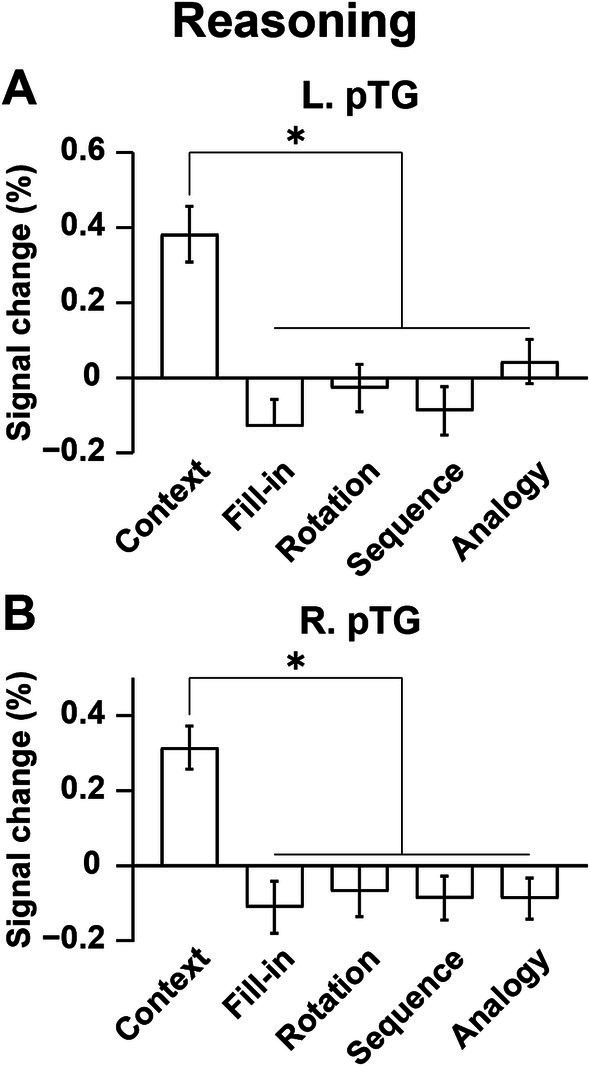
Bilateral pTG activations selective to the Context condition. **(A)** Signal changes in the L. pTG. Activations were significantly higher under the Context condition than the other four combined conditions. **(B)** Signal changes in the R. pTG, which replicated those of the L. pTG. The error bars denote the SEM. *p < 0.05.

We further focused on the L. pTG (Figure 7A) and R. pTG (Figure 7B), whose ROIs were chosen from the activated regions in the [Sign (Story) − Counting] contrast (see Figure 6A), determined independently from the Reasoning task. In both ROIs, a significant main effect of condition was indicated for signal changes (rANOVA, *p* < 0.0001). Moreover, the activations were significantly higher under Context than the other four combined conditions (L.: *t*[17] = 9.7, *p* < 0.0001; R.: *t*[17] = 11.8, *p* < 0.0001), demonstrating that the activations had selectivity only for Context.

## Discussion

To examine commonalities that underlie reasoning and language, we prepared illustrative quizzes for the Reasoning task (Figure 1). We also used story videos in JSL for the Sign task (Figure 2A). Brain activations were examined for native signers of JSL, and the following results were obtained. First, in the [Context − Fill-in] contrast, multiple regions were activated, including language areas such as the L. LPMC and L. IFG (Figure 4A). By using conjunction and ROI analyses (Figures 4E,F,5), we clarified two distinct systems, which were differentially recruited under the Sequence and Analogy conditions: the *recursive* system (L. LPMC/dIFG and R. LPMC) and the *propositional* system (L. IFG), respectively (see Table 1). Secondly, we identified activations during Sign in the L. LPMC, L. IFG, and other temporal regions (Figures 6A,B). Moreover, regarding contextual comprehension processes during Sign (Story), we found that the L. IFG and bilateral pTG were commonly activated between Sign (Story) and Context (Figure 6C). Thirdly, in the bilateral pTG, activations were selective only under the Context condition and not under the other four conditions (Figure 7), confirming its role as the *clausal* system (see Table 1). We successfully identified three critical systems for both language and thought processes. The latter reasoning processes are thus tightly coupled with linguistic operations that are more fundamental and deemed to be simplest operations (Chomsky et al., 2023).

Regarding the *recursive* system, we have shown that the structural depth of sentences best accounted for activations in the bilateral LPMC (Umejima et al., 2023) and L. dIFG (Ohta et al., 2013) by using syntactic decision tasks. As regards the *propositional* system, ventral L. IFG activations were identified in discourse level judgments (Homae et al., 2002; Inubushi and Sakai, 2013), as well as in subject-verb matching for a newly acquired language (Umejima et al., 2021a). The L. F3op/F3t (including the L. dIFG), L. LPMC, and L. F3t/F3O (i.e., ventral portion of the L. IFG) have been shown to play different roles in the three syntax-related networks, i.e., networks I, II, and III (Kinno et al., 2014). These left frontal regions were also recruited in the present Sign task. Moreover, the R. LPMC and R. F3t/F3O (including the R. IFG shown in Figure 6B) belong to networks I and III, respectively (Tanaka et al., 2020). Furthermore, the R. pTG and L. pTG, which were also recruited in the Sign task, belong to networks I and III, respectively (Kinno et al., 2014). In addition to syntax, the networks I, II, and III subserve its supportive system, input/output interface (including motor sequencing, planning, and imagery), and semantics (including semantic memory), respectively. These regions are thus integrated into the syntax-related networks, revealing the existence of common neural substrates for both language and thought.

There has been a serious debate between localist and holist views since the earliest lesion studies (Rutten, 2022). While some researchers claim that the language network lacks selectivity to lexico-semantics or syntax (Fedorenko et al., 2020), this argument may be due to the use of tasks assessing pragmatics rather than syntax or specific operations. On the other hand, a recent fMRI data study with artificial models of language has shown dissociation between lexico-semantics and syntax (Pasquiou et al., 2023). Converging studies are needed to elucidate functional localization of syntactic and semantic processes.

In a larger-scale study with deaf signers (*N* = 28), we have previously shown that activated regions in the left frontal language areas gradually expanded in the dorso-ventral axis, corresponding to a difference in linguistic units at the word, sentence, and discourse levels (Inubushi and Sakai, 2013). These regions were consistently the key regions in the present study, showing that hearing status did not affect our results. Moreover, we have previously established that the activation patterns of deaf signers, hearing signers, and hearing non-signers were essentially identical during sentence processing in JSL/JPN (Sakai et al., 2005), indicating commonality among languages in different modalities. Our previous studies have further demonstrated that the L. IFG is activated for various syntactic tasks not only in the first language but also in the second language (Sakai, 2005), as well as in the third and fourth languages (Umejima et al., 2024). Future functional imaging studies on humans should shed light on universal and fundamental aspects of language and thought, beyond the superficial differences in hearing status, modalities, and languages.

## Data Availability

The raw data supporting the conclusions of this article will be made available by the authors, without undue reservation.

## Footnotes

^1^ http://www.fil.ion.ucl.ac.uk/spm/

^2^ http://www.gin.cnrs.fr/en/tools/aal/

^3^ http://Neuromorphometrics.com/

^4^ http://marsbar.sourceforge.net/

^5^ https://www.r-project.org/

^6^ http://www.neuropowertools.org/neuropower/neuropowerstart/
